# Supplementary material for: Indoor aeroallergens from American cockroaches and mites initiate atopic march via cutaneous contact in a murine model
Source: PLoS One. 2023 Jul 27;18(7):e0289138. doi: 10.1371/journal.pone.0289138 (PMC10374041; doi:10.1371/journal.pone.0289138)
Supplement: S1 Table — (DOCX) [file pone.0289138.s001.docx]

**Supporting Information**

**Supplementary Table S1. The sequences of murine gene-specific primers used in real-time PCR**

| **Gene (mouse)** | | **Sequences** | **Product size (bp)** |
| --- | --- | --- | --- |
| **IL-4** | F | 5’ AGC CAT ATC CAC GGA TGC GAC AAA 3’ | 176 |
|  | R | 5’ AAT ATG CGA AGC ACC TTG GAA GCC 3’ |  |
| **IL-5** | F | 5’AGCACAGTGGTGAAAGAGACCTT 3’ | 117 |
|  | R | 5’ TCCAATGCATAGCTGGTGATTT 3’ |  |
| **IL-9** | F | 5′-AACAGTCCCTCCCTGTAGCA-3′ | 110 |
|  | R | 5′-AAGGATGATCCACCGTCAAA-3′ |  |
| **IL-13** | F | 5’ AGA CCA GAC TCC CCT GTG CA 3’ | 123 |
|  | R | 5’ TGG GTC CTG TAG ATG GCA TTG 3’ |  |
| **IFN-γ** | F | 5’ GGC CAT CAG CAA CAA CAT AAG CGT 3’ | 118 |
|  | R | 5’ TGG GTT GTT GAC CTC AAA CTT GGC 3’ |  |
| **TNF-α** | F | 5’ CAT CTT CTC AAA ATT CGA GTG ACA A 3’ | 175 |
|  | R | 5’ TGG GAG TAG ACA AGG TAC AAC CC 3’ |  |
| **IL-10** | F | 5’ CCA AGC CTT ATC GGA AAT GA 3’ | 155 |
|  | R | 5’ AGG GGA GAA ATC GAT GAC AG 3’ |  |
| **MCP-1** | F | 5'-GACCCGTAAATCTGAAGCTAATGC-3' | 118 |
|  | R | 5'-AATTAAGGCATCACAGTCCGAGTC-3' |  |
| **IL-31** | F | 5’ CAG CTG TTT CAA CCC ACT G 3’ | 121 |
|  | R | 5’ CAG TTC TGC CAT GCA GTT TG 3’ |  |
| **TSLP** | F | 5’-GGA GAT TTG AAA GGG GCT AAG -3’ | 168 |
|  | R | 5’- TGG GCA GTG GTC ATT GAG- 3’ |  |
| **β-actin** | F | 5’ GGC CAA CCG TGA AAA GAT GA 3’ | 251 |
|  | R | 5’ CAC GCT CGG TCA GGA TCT TC 3’ |  |
